# Diet in Brain Health and Neurological Disorders: Risk Factors and Treatments

**DOI:** 10.3390/brainsci9090234

**Published:** 2019-09-13

**Authors:** Jason Brandt

**Affiliations:** Johns Hopkins University School of Medicine, Baltimore, MD 21205, USA; jbrandt@jhmi.edu

The role of nutrition in health and disease has been appreciated from time immemorial. Around 400 B.C., Hippocrates wrote: “Let food be thy medicine and medicine be thy food.” In the 12^th^ century, the great philosopher and physician Moses Maimonides wrote “any disease that can be treated by diet should be treated by no other means.” Now, in the 21^st^ century, we are bombarded by claims in the media of “superfoods,” wondrous nutritional supplements, and special diets that promise to cure or prevent disease, improve health, and restore functioning. Much of the focus has been on neurological disease, brain health, and psychological functioning (behavior, cognition, and emotion).

The hyperbole aside, the past two decades have seen considerable progress in our understanding of the role of specific nutrients and dietary patterns to brain development, physiology, and functioning [1,2,3,4]. The chapters in this volume are but a sampling of the latest research on the role of specific compounds and nutrients in brain function and dysfunction, and use of diet for the prevention and treatment of neurological and psychological disorders.

The *ω*-3 and *ω*-6 polyunsaturated fatty acids (PUFAs) have long been recognized as essential to cell membranes and normal neuronal function. Deficiencies *ω*-3 PUFAs have been associated with everything from mood disorders to schizophrenia to Alzheimer’s disease. Fuentes-Albero and colleagues [5] report that Spanish schoolchildren with attention-deficit hyperactivity disorder (ADHD) consume diets that are lower in *ω*-3 PUFAs than their peers without ADHD. Since this is a case-control observational study rather than a randomized trial, no causative conclusions can be drawn. However, the authors’ call for increased consumption of fatty fish (the main dietary source of *ω*-3 PUFAs) as a component of healthy eating patterns is well supported by other research.

Another constituent of just about everyone’s diet is caffeine, by far the most commonly used psychoactive substance worldwide. Ueda and Nakao [6] examined the acute effects of this stimulant on cognition and electrophysiology in a small group of healthy young men. To achieve peak blood level quickly, the drug was “vaped” rather than ingested. Caffeine produced a slightly greater increase in working memory performance (N-back task) than did placebo. EEG power in the theta band was enhanced after inhaling caffeine vapor, but only from selected right-hemisphere frontal, central, and temporal electrodes. The authors conclude that caffeine, an adenosine receptor blocker, increases the activity of right-hemisphere regions that mediate attentional and executive functions required for the working memory task. Furthermore, transpulmonary administration of caffeine resulted in a very rapid change in brain activity. Its effects on other aspects of cognition and emotional states remain to be investigated.

One of the most active areas of nutritional research is the role of dietary fats on brain functioning. Loprinzi and colleagues [7] surveyed the scientific literature on the effects of high-fat diets on learning and memory. They found that all laboratory studies of rodents found significant deleterious effects of high-fat diets compared to standard diets. However, all reviewed studies also found that having subjects engage in regular physical exercise could counteract this effect. A variety of mechanisms are proposed, including increases in BDNF and synapsin-1 and decreases in proinflammatory cytokines and insulin resistance. Whether such a beneficial effect would be seen for all varieties of human memory (e.g., episodic/semantic, declarative/procedural, retrospective/ prospective) after an exercise regimen (of what type? how vigorous? how often?) remains to be determined.

Speaking of fats, there is at least one form of high-fat diet that has clear neurotherapeutic and perhaps neuroprotective effects. As described by McDonald and Cervenka [8], the ketogenic diet, which combines large amounts of fat with very low amounts of carbohydrates, induces the liver to produce ketone bodies (acetoacetate and β-hydroxybutyrate) which are then used as a source of energy for neurons. Ketogenesis also alters the balance of excitatory and inhibitory neurotransmitters, modifies gene expression, reduces oxidative stress and inflammation, and has other effects on brain function. The ketogenic diet was introduced for the treatment of epilepsy 100 years ago, and is today a pillar in the treatment of medication-refractory seizures. It is currently being investigated for a wide variety of other neurological disorders, including stroke, glioblastoma, amyotrophic lateral sclerosis, and Alzheimer’s disease. 

In the final contribution to this volume, Poulimeneas and colleagues [9] tackle a vexing question in nutritional neuroscience, namely the brain mechanisms that support successful weight loss in obese people. They review the literature on functional neuroimaging in weight-loss maintainers compared to currently obese and lean individuals. Although only eight studies, using very different methods, were located, some trends were discerned. Formerly-obese people appear to display the same cerebral activation to food stimuli in reward-related brain regions as do obese people. However, they also display heightened activation in regions of the prefrontal cortex associated with inhibitory control. The authors then speculate on the biological mechanisms underlying this “neural restraint” among weight-loss maintainers. Since most studies have been cross-sectional rather than longitudinal, we do not know whether these brain activation patterns are contributors to or consequences of successful weight-loss maintenance. One way to address this might be to determine whether those who lose and maintain a great deal of weight via bariatric surgery would display the same cerebral activation patterns as those who achieved their weight loss the “old-fashioned way” (diet, exercise, and behavior modification).

This slim volume cannot do justice to the richness of the evolving neuroscience related to diet and nutrition. I suspect that the next two decades will see even more exciting advances. Stay tuned.
